# Pediatric Delirium Educational Tool Development With Intensive Care Unit Clinicians and Caregivers in Canada: Focus Group Study

**DOI:** 10.2196/53120

**Published:** 2023-12-11

**Authors:** Michael Wood, Kavi Gandhi, Andrea Chapman, Peter Skippen, Gordon Krahn, Matthias Görges, S Evelyn Stewart

**Affiliations:** 1 BC Children’s Hospital, Vancouver, BC, Canada; 2 Department of Psychiatry, University of British Columbia, Vancouver, BC, Canada; 3 Department of Pediatrics, University of British Columbia, Vancouver, BC, Canada; 4 Department of Anesthesiology, Pharmacology, and Therapeutics, University of British Columbia, Vancouver, BC, Canada; 5 BC Mental Health and Substance Use Services Research Institute, Vancouver, BC, Canada

**Keywords:** pediatric delirium education, pediatric ICU, focus groups, prototyping, end users, users, education, educational, educational tool, tool, development, caregiver, Canada, PICU, pediatric intensive care unit, quality of life, child, children, family resource, cognition, clinical utility, intensive care unit

## Abstract

**Background:**

Pediatric intensive care unit (PICU)–associated delirium contributes to a decline in postdischarge quality of life, with worse outcomes for individuals with delayed identification. As delirium screening rates remain low within PICUs, caregivers may be able to assist with early detection, for which they need more education, as awareness of pediatric delirium among caregivers remains limited.

**Objective:**

This study aimed to develop an educational tool for caregivers to identify potential delirium symptoms during their child’s PICU stay, educate them on how to best support their child if they experience delirium, and guide them to relevant family resources.

**Methods:**

Web-based focus groups were conducted at a tertiary pediatric hospital with expected end users of the tool (ie, PICU health care professionals and caregivers of children with an expected PICU length of stay of over 48 h) to identify potential educational information for inclusion in a family resource guide and to identify strategies for effective implementation. Data were analyzed thematically to generate requirements to inform prototype development. Participants then provided critical feedback on the initial prototype, which guided the final design.

**Results:**

In all, 24 participants (18 health care professionals and 6 caregivers) attended 7 focus groups. Participants identified five informational sections for inclusion: (1) delirium definition, (2) key features of delirium (signs and symptoms), (3) postdischarge outcomes associated with delirium, (4) tips to inform family-centered care, and (5) education or supportive resources. Participants identified seven design requirements: information should (1) be presented in an order that resembles the structure of the clinical discussion around delirium; (2) increase accessibility, recall, and preparedness by providing multiple formats; (3) aim to reduce stress by implementing positive framing; (4) minimize cognitive load to ensure adequate information processing; (5) provide supplemental electronic resources via QR codes; (6) emphasize collaboration between caregivers and the health care team; and (7) use prompting questions to act as a call to action for caregivers.

**Conclusions:**

Key design requirements derived from end-user feedback were established and guided the development of a novel pediatric delirium education tool. Implementing this tool into regular practice has the potential to reduce distress and assist in the early recognition and treatment of delirium in the PICU domain. Future evaluation of its clinical utility is necessary.

## Introduction

### Background

Delirium is a neurological dysfunction characterized by an acute onset of inattention, consciousness fluctuations, or disorganized thinking [1,2]. Approximately 25% of pediatric intensive care unit (PICU) patients experience delirium throughout their stay [3], with greater prevalence among children who require mechanical ventilation (54%-74%) [4]. PICU patients with delirium tend to have an increased length of stay, and pediatric delirium has been independently associated with mortality [5]. PICU survivors frequently experience substantial physical and psychosocial morbidities, such as sleep disturbances, anxiety, depression, and memory impairments, which may increase in severity with increased delirium duration [6,7]. Following discharge, PICU patients with delirium also experience decreased quality of life (eg, physical functioning, bodily pain, and social behaviors) [8], as do their caregivers (eg, physical and emotional well-being [8] and financial difficulties [6]). Collectively, pediatric delirium results in long-term complications affecting both patients and their caregivers.

An educational tool can help caregivers recognize delirium symptoms, aid in delirium detection, and help diminish caregiver anxiety and distress when delirium occurs [4,9-18]. Early intervention has been associated with a substantial decrease in delirium duration and subsequent complications [19], further indicating the need to use the caregiver’s unique and vital role in recognizing deviations from their child’s premorbid baseline functioning [9,14]. Family-based identification can be comparable to clinical identification [14] and is essential in pediatrics, given that only 2% to 7% of PICU patients are routinely screened for delirium [4]. Thus, empowering caregivers to increase their understanding and involvement in preventing, detecting, and managing delirium has been suggested [9,12-14,20,21]. Despite these recommendations and the clinical relevancy of delirium, family caregiver education remains rare [10], representing an opportunity to implement practical educational tools in the PICU.

### Objectives

We aimed to develop an educational tool for caregivers whose child may develop PICU-associated delirium, which would enable them to identify signs of delirium and bring that information to the health care providers’ attention during the PICU stay, provide education on how they can best support their child if their child experiences delirium, and provide relevant resources to support families.

## Methods

### Study Design

We applied patient-oriented research principles [22]. We conducted a qualitative study using focus groups with caregivers (ie, parents of children currently receiving, or who recently received, pediatric intensive care with an expected length of stay for over 48 h) and clinicians or allied health professionals (eg, pediatricians, psychiatrists, clinical fellows, resident physicians, psychologists, nurse practitioners, nurses, and pharmacists), all of whom have experience in PICU-associated delirium and who work at BC Children’s Hospital (BCCH) in Vancouver, British Columbia, Canada.

### Ethical Considerations

Ethical approval was obtained from the University of British Columbia and Children’s & Women’s Health Centre of British Columbia Research Ethics Board (H22-03478; date of approval: February 28, 2023; principal investigator: SES). Our findings are reported following the COREQ (Consolidated Criteria for Reporting Qualitative Research) checklist [23].

### Participant Recruitment and Eligibility

Clinicians and allied health professionals were approached by trained research staff at BCCH and contacted via departmental email distribution lists. In contrast, caregivers were recruited in person in the PICU during their child’s hospital stay. After a trained research team member described the study and answered any questions, informed consent was obtained in person or electronically using Research Electronic Data Capture (REDCap; Vanderbilt University) [24,25]. As the focus groups were conducted over the web, participants were required to have an internet connection and access to an electronic device (eg, tablet, smartphone, or computer). To encourage participation, participants were provided CAD $25 (US $18.35) per hour as an honorarium for their time and expertise. Each focus group session aimed to have mixed participant types and included approximately 3 to 5 clinicians and allied health professionals and 3 to 5 caregivers.

### Data Collection

Following informed consent, a brief demographics questionnaire (eg, age range, education level, etc) was administered using REDCap [25]. Two trained research team members (MW and KG) conducted the focus group meetings between February and August 2023 using Zoom (Zoom Video Communications). One researcher facilitated the sessions (MW) while another research team member took notes (KG); only the 2 research team members and the recruited participants attended each session. At the start of each focus group, the team members introduced themselves and their role and then asked the participants to provide a brief introduction. A team member gave an overview of the research program, including the rationale for educating caregivers about pediatric delirium.

The focus groups were iterative, consisting of 2 stages, and participants were invited to both. First, sessions used guided questions to prompt participants to discuss three themes: (1) how delirium education has been given to caregivers previously, or how clinicians or allied health professionals have previously provided delirium education; (2) what educational tools or instruments participants have used; and (3) what type of information is pertinent to educate caregivers about pediatric delirium (see Multimedia Appendix 1). We then shared adult delirium educational tool examples (created or adapted from Vanderbilt University) to elicit design requirements and visualization preferences to inform prototype development. In follow-up sessions, we reviewed the findings from the previous focus groups and screen-shared our educational tool prototype to obtain end-user feedback to guide the final design. While viewing examples and the prototype, participants were prompted to indicate their general thoughts on the designs, such as what they liked or disliked about the design and suggestions for improvement.

Sessions lasted approximately 1 hour, were audio recorded, and were digitally transcribed using the live transcription function in Zoom. Transcripts were verified by a research team member (MW or KG), and participant names were replaced by sequential identifiers. For methodological rigor, our data saturation criterion [26] aimed to discontinue data collection when we reached informational redundancy. Specifically, 2 research team members (MW and KG) determined that similar comments and concerns were repeatedly discussed across sessions and that data saturation had occurred.

### Data Analysis

Participant characteristic questionnaire data were summarized using R software (version 4.3.1; R Foundation for Statistical Computing). Qualitative data (ie, focus group transcripts) were analyzed using NVivo (QSR International) and summarized using thematic analysis [27]. Two research team members (MW and KG) independently reviewed 2 transcripts and used inductive coding [28] to develop a preliminary list of thematic codes organized by theme, subtheme, and participant labels to describe these data and generated a preliminary codebook [29]. To ensure consistency, these researchers then compared interpretations, resolved any discrepancies, and applied these codes to the remaining transcripts using deductive coding [28]. To ensure key concepts were not missed and that additional coding remained consistent, these researchers iteratively discussed additional themes that emerged after coding the remaining transcripts and adjusted the coding framework accordingly.

Coded quotes were organized by a theme, subtheme, and participant type (ie, a clinician or allied health professional [denoted by “HCP”] or a caregiver [denoted by “CG”]). Prominent themes that emerged from focus groups (see the *Results* section) were used to generate requirements to develop the delirium education tool. Participant responses to the open-ended questions defined how delirium education is conducted in practice and what sections should be included in the tool to resemble this discussion; their responses also suggested potential design requirements to ensure effective use in hospitals, which were further explored based on participant feedback on the adult delirium education tools.

## Results

### Focus Group Participant Demographics

In all, 24 participants, including 18 PICU clinicians and allied health care professionals (6 registered nurses, 3 psychologists, 3 clinical fellows, 4 psychiatrists, 1 physiotherapist, and 1 intensivist) and 6 family members, attended 7 focus group sessions consisting of 4 to 6 participants (1 session was comprised of only 2 participants due to cancellations), and 57% (4/7) of the sessions consisted of mixed groups (combining clinicians, allied health care professionals, and caregivers). When approached in the PICU, 2 family members declined because they did not have the time and energy to participate, 11 family members could not be contacted following informed consent, and no participants were excluded. However, 67% (4/6) of the caregivers and 40% (6/15) of the health care professionals dropped out of the study from stage 1 to stage 2; thus, 3 additional health care professionals were recruited during stage 2 accordingly. These high attrition rates were predominately attributed to a child being readmitted (worsening or additional illness) or limited availability. Of the enrolled participants, 79% (19/24) identified as female, and 88% (21/24) were aged <50 years. Of the family member participants, 67% (4/6) had a high school diploma (or equivalent) and 33% (2/6) had either a certificate (university or nonuniversity) or university degree.

### PICU Delirium Education in Practice: Key Themes

Data from focus group discussions were grouped into 3 thematic domains, described in detail below, with a summary of design requirements and tool informational sections shown in Tables 1 and 2, respectively.

**Table 1.** Summary of themes and design requirements identified from the initial focus groups with health care professionals and caregivers of critically ill children.

Identified themes	Design requirements
Present tool information in a logical order	R1.1: Present educational information in an order that resembles the structure of the discussion between health care professionals and caregivers around delirium
Ensure that the tool is user-friendly	R2.1: Provide multiple formats to increase information accessibility and recall, and to ensure that all families feel prepared R2.2: Minimize potential distress by implementing positive framing R2.3: Reduce cognitive load of caregivers to ensure effective information processing R2.4: Make detailed supplemental electronic resources readily available via web links and QR codes
Delirium education should provide a sense of agency	R3.1: Emphasize the importance of collaboration between the caregiver(s) and the health care team R3.2: Ask prompting questions to act as a call to action for the caregiver(s)

**Table 2.** Summary of informational sections that should be included in the tool to ensure effective delirium education.

Section	Description
S1	Provide a succinct delirium definition to indicate that it is a common and transient condition among critically ill children
S2	Describe common signs and symptoms to ensure that families can identify key features of pediatric delirium
S3	Highlight clinically relevant long-term outcomes associated with delirium to contextualize the importance of early detection and management
S4	Indicate suggestions on how caregivers can assist with the care of their child who is currently experiencing delirium
S5	Incorporate education and supportive resources for families that require additional information or assistance
Additional sections for consideration	Delirium risk factors, potential causes, and mental health and supportive resource for caregivers

#### Overview of Delirium Education in Practice

When considering how clinicians typically provide delirium education to caregivers, PICU health care professionals indicated that they do not typically discuss delirium until “staff start to observe symptom onset” (HCP09). As many health care professionals outside of psychiatry and psychology do not feel that they have delirium expertise, clinicians will typically “provide a short summary” (HCP08) and discuss delirium broadly (eg, signs and symptoms, potential causes, and long-term outcomes) following onset. During this discussion, clinicians may also describe delirium management techniques (eg, prompt extubation, sedation vacation, and early mobilization) if prompted by caregivers. As “parents are typically distressed” (HCP08) during their child’s PICU stay and feel that they “lack control for caring for their child” (HCP01), health care professionals consistently provide details on how the family can assist their child (eg, bringing the child’s favorite toy or blanket, family photos, and art). Subsequently, the discussion concludes by ensuring that the family has an accurate understanding of delirium and answering any remaining questions. Caregivers who had a child experience delirium indicated a similar process, whereas caregivers of children without delirium believed that the process and format would be informative (requirement R1.1 in Table 1).

#### Previous Experience With Educational Tools or Instruments

Most participants indicated that delirium education is a conversation at the bedside and that tools or instruments should be regularly implemented. Some clinicians indicated they might refer caregivers to the American Academy of Child & Adolescent Psychiatry web-based resources, such as “Delirium in Children and Adolescents” or “When Your Child has Pediatric Delirium.” Notably, most health care professionals were unaware that delirium education tools existed and indicated that their clinic or unit “doesn’t currently have a delirium pamphlet or tool” (HCP03) that they can refer families to. A caregiver indicated that they had used surgical education tools (eg, websites and brochures) previously, which were “extremely helpful” (CG02), acted as a resource that could be reviewed as required, and ensured that they were prepared for the surgical journey (requirement R2.1). Another caregiver echoed this sentiment and indicated that that during a stressful event, such as having a child in the PICU, “you don’t necessarily remember what was said [by health care professionals]” and having “this information to refer back to is paramount” (CG06) to ensuring effective delirium education.

#### Information Required to Provide Effective Delirium Education in the PICU

First, participants agreed that delirium should be clearly defined. Due to potential delirium-associated distress, participants suggested that the tool should then provide contextual information to emphasize that delirium is a commonly occurring condition among critically ill children (eg, 1 in 4 children experience delirium) and is a “temporary condition” (HCP12) that typically “resolves as the child’s health improves” (HCP10; section S1 in Table 2).

As parents may be able to assist with the early detection of “subtle changes in their child’s behavior [from baseline]” (HCP11), participants indicated that a “signs and symptoms” (HCP12) section should identify common features of delirium (eg, fluctuating course, confusion, altered sleep-wake cycle, rapid mood changes, hypoactive to hyperactive features, etc; section S2). Most participants further indicated that a section on outcomes associated with delirium (eg, increased length of hospital stay and decreased postdischarge quality of life) would contextualize the importance of prompt delirium detection and management (section S3). Some clinicians believed that the tool should indicate potential risk factors associated with delirium, as well as indicate how children are diagnosed and treated; however, most participants believed that indicating risk factors may “increase family anxiety” (HCP14). Participants further suggested that due to the complexity of the PICU and the stress associated with their child’s acute illness, the educational tool should apply positive framing throughout (requirement R2.2).

To ensure caregivers are effectively used in early recognition of delirium and helpful treatment strategies, participants indicated that the tool should emphasize “the collaborative relationship between families and the medical team” (HCP10; requirement R3.1) and indicate how caregivers can “have an active role in their child’s care” (HCP06; section S4). For example, parents could bring “comfort items” (CG02) to the hospital, as well as implement daily routine reminders, such as opening the blinds, turning on room lights during the day, or brushing their child’s teeth and showering them when it is safe to do so.

Most participants indicated that the education tool should conclude with a section for additional resources, such as pediatric delirium websites and options for local supportive services (section S5). As having a child admitted to the PICU greatly “impacts parents and their own mental health” (CG06), some participants further indicated that mental health and supportive resources should be available to ensure that caregivers feel supported during their child’s hospital stay.

### Additional Requirements and Suggestions Identified From Reviewing Adult Delirium Education Tools

#### Minimize Cognitive Load of Users

Participants identified that education tools may be cognitively demanding for caregivers who are already experiencing the distress associated with having a critically ill child (requirement R2.3); therefore, the tool should serve as a “quick reference” that is “a summary of the clinical discussion” (HCP11). Specifically, the tool should (1) implement “lay language [throughout]” (CG05); (2) avoid redundancy; (3) have “clear separation between each section” (HCP14); (4) use “bullet points to ensure information is easier to process” (CG06); and (5) use informative and representative icons. Participants further indicated that the 1-page format was less cognitively demanding overall and more accessible to review as an electronic resource than a trifold brochure, but a more detailed document may be beneficial for some families.

#### The Delirium Education Tool Should Use Prompting Questions

Although the tool can only provide generic pediatric delirium education, most participants felt that the tool should provide a sense of personalization. The tool should “center on the child” (HCP11) by using “prompting questions” (HCP04) to act as a call to action for parents (eg, Is your child experiencing any of the following? and How can you help your child while they experience delirium?), which would engage families and potentially provide a sense of agency over their child’s care (requirement R3.2).

#### Include Multiple Formats to Increase Use

Most participants believed the tool should have a 1-page version and a more detailed version readily available. Participants requested that the 1-page tool should remain a physical copy that provides only “necessary information” to ensure “its education information is not overwhelming” (CG02) and that families can read it “when they have time to process the information” (HCP09). Families (and potentially staff) that request additional educational information should have the option to access a more detailed document and other delirium-related resources via web links or QR codes (requirement R2.4).

### Additional Suggestions to Finalize the Delirium Education Tool Design

**Figure 1.**
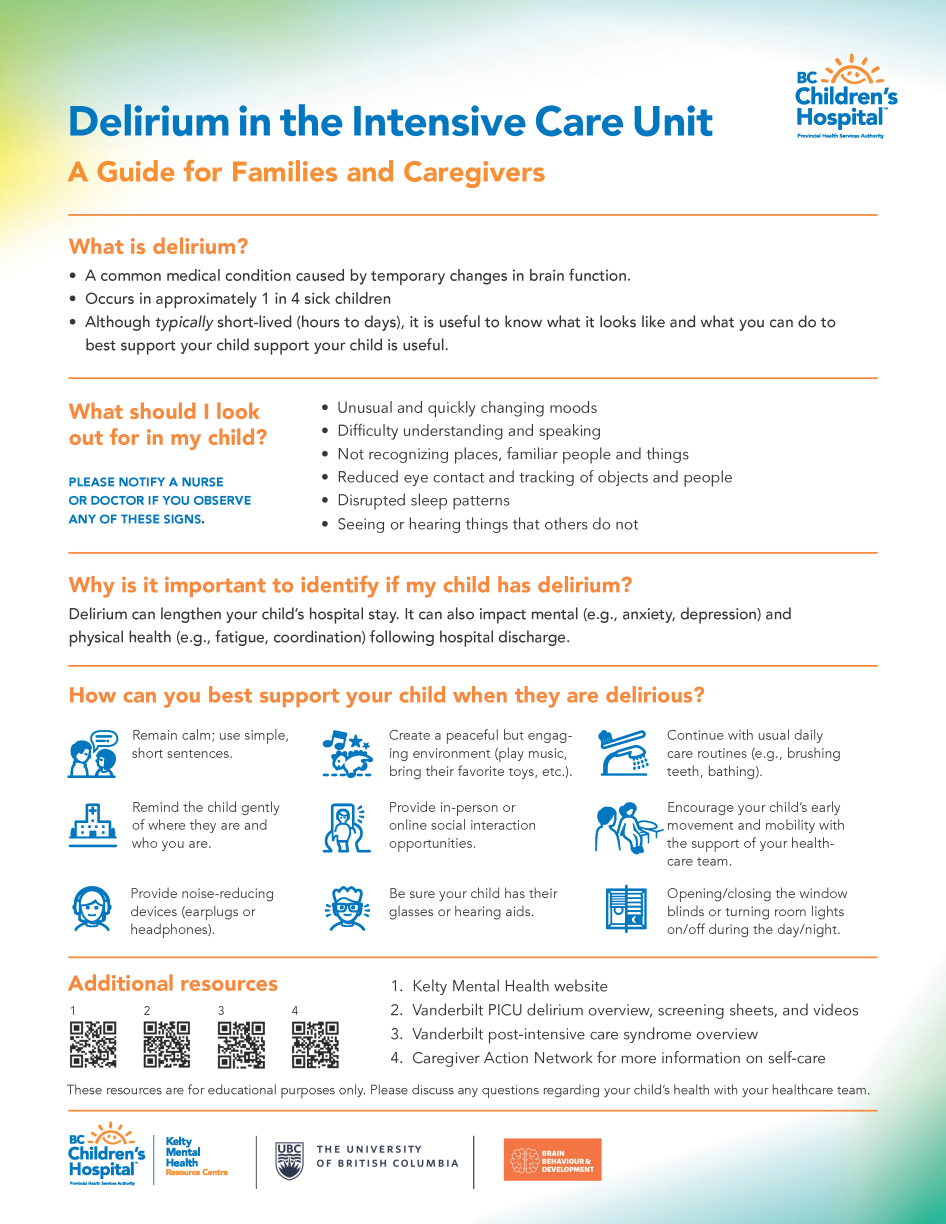
One-page pediatric intensive care unit (PICU) delirium educational tool for caregivers.

**Figure 2.**
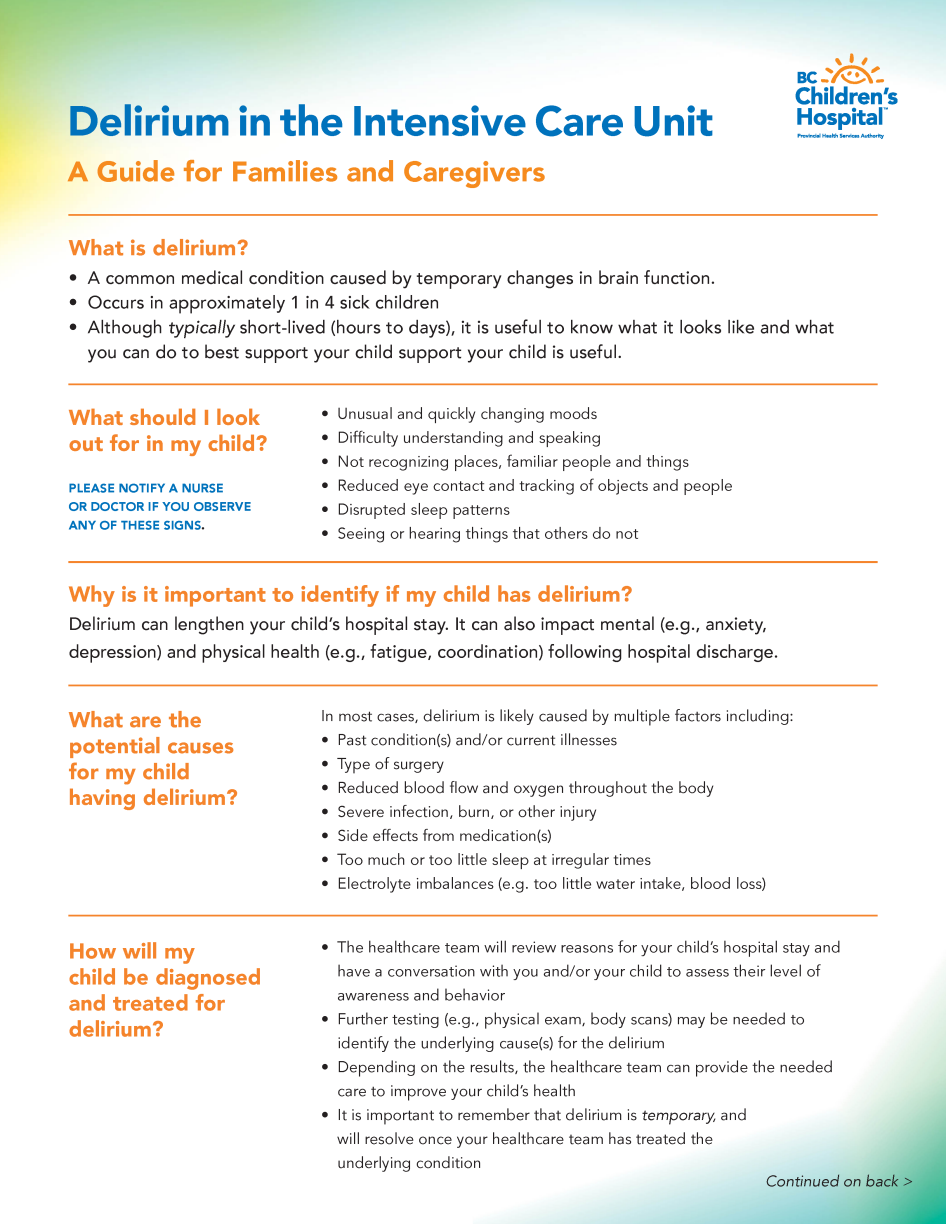
Two-page (front) pediatric intensive care unit (PICU) delirium educational tool for caregivers.

**Figure 3.**
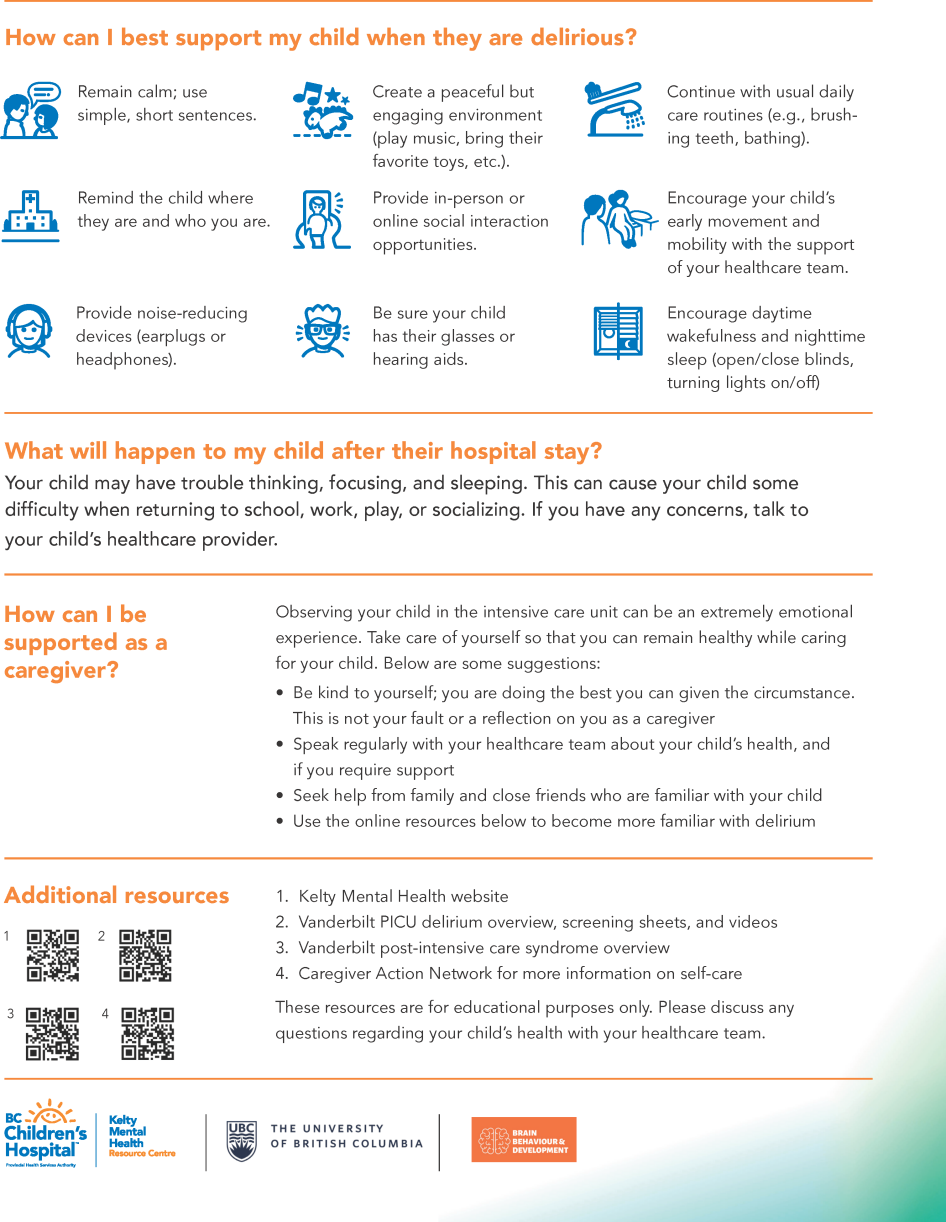
Two-page (back) pediatric intensive care unit (PICU) delirium educational tool for caregivers.

Participants indicated that our delirium education tool design (Figures 1-3) effectively included all sections and requirements identified from the previous sessions and that the tool would be highly beneficial to future families whose child is experiencing delirium in the PICU. While viewing the prototype, end users provided minor suggestions for improvement and indicated that the tool should (1) implement bold or italic text to emphasize key concepts; (2) provide section headings for each piece of text (acting as a question and answer format); (3) reduce medical terminology, vague constructs (eg, irregular moods), and redundant text; (4) include informative and representative icons; (5) ensure lists have a logical flow; and (6) contextualize if symptoms are in hospital versus after discharge. These suggestions were directly incorporated into our prototype, resulting in the finalized design of a multiformat delirium education tool in pediatrics.

## Discussion

### Primary Findings

During the focus group sessions, our expected end users (health care professionals and caregivers) indicated five informational sections to develop a pediatric delirium education tool: (1) delirium definition, (2) key features (signs and symptoms), (3) postdischarge outcomes associated with PICU delirium onset, (4) tips and suggestions to inform family-centered care, and (5) education or supportive resources. To use educational information in practice, participants further indicated seven design requirements: information should (1) be presented in an order that resembles the structure of the clinical discussion around delirium; (2) increase accessibility, recall, and preparedness by providing multiple formats; (3) reduce stress by implementing positive framing; (4) minimize the cognitive load of users to ensure adequate information processing; (5) provide supplemental electronic resources via QR codes; (6) emphasize collaboration between caregivers and the health care team; and (7) ask prompting questions to act as a call to action for caregivers. These findings culminated in the development of a delirium education tool for the PICU.

### Comparison With Prior Work

Previous research in adult palliative care has resulted in educational tools with similar informational sections and requirements: for example, including a delirium definition, causes, signs and symptoms, as well as the treatment of delirium [11,15,17,18]; implementing easily understood language throughout [18]; using direct, specific, and action-oriented lists [18]; applying simple layouts to ease processing; reducing text [11,15,17,18], and applying intuitive designs (eg, bold contrasting colors and large headings) [18]. Additional suggestions included implementing rest periods to mitigate caregiver exhaustion and daily communications with the patients’ health care professionals about delirium [15]. We tried to address these suggestions by including additional sections on how caregivers can be supported and emphasizing collaboration between caregivers and the health care team. These previous studies were conducted in adult populations, and these findings broadly agree with those from our study. However, previous delirium education tool developments have rarely implemented patient-oriented research principles [22], such as directly involving expected end users [11,18], which may limit their implementation and clinical utility.

Adult delirium education tools have increased caregiver understanding of the causes of delirium [17], and health care professionals indicated that they were an efficient way to support caregivers and facilitate their involvement in providing care [11]. Such educational tools have led caregivers to report increased comfortability in delirium discussions with other family members, increased confidence in caretaking abilities, and decreased emotional distress (eg, feeling responsible, guilty, and powerless) during delirium episodes [15]. Furthermore, caregivers who received a delirium educational tool reported lower levels of anxiety and depression compared to caregivers who did not [30]. Usability surveys further indicated that health care professionals and caregivers found adult delirium education tools to be comprehensive and easily understandable [11] and that health care professionals intended to use them with future families [18]. Taken together, delirium education tools have the potential to increase caregivers’ knowledge and confidence while decreasing their delirium-related distress, which suggests that future research is warranted to demonstrate both tool validity and usability in pediatrics.

### Limitations

Our participants comprised a representative cohort of PICU health care professionals but only a small number of caregivers from a single center, which may limit the transferability of our findings. We also had a large amount of attrition, which may have further limited transferability but also reflects the challenges of conducting qualitative research with repeated observations among complex patient populations. Future studies will need to implement multiple strategies (eg, providing incentives, conducting rapport-building exercises, enacting frequent communication, and indicating study benefits) to improve retention [31]. However, we included a wide range of health care professions (eg, psychiatrists, pharmacists, nurses, etc) and achieved data saturation; thus, robust findings were likely identified. To reduce additional distress and facilitate effective instruction, including children in future focus groups may be imperative; however, due to the large number of patients aged <7 years at our site, parents and caregivers were deemed as an appropriate proxy. Despite language interpretation services and closed captioning being offered during recruitment, our focus groups comprised only English-speaking participants, which may have further limited transferability. Although participants provided feedback on our current prototype, these findings lay the foundation to investigate the clinical impact of delirium education and inform the development of a study to investigate the family’s role in detecting delirium in pediatrics. Assessing the clinical utility and usability of our pediatric delirium education tool prototype was beyond the scope of this study due to limited resources, and further research is warranted.

### Conclusions

Our study identified several requirements for developing a PICU-associated delirium education tool, such as presenting the information in a way that resembles the consult, providing multiple formats, implementing positive framing, minimizing the cognitive load of users, using QR codes for additional resources, emphasizing collaboration, and asking prompting questions to act as a call to action. Although clinical evaluation is still required, implementing an educational tool guided by clinicians, allied health professionals, and caregivers into clinical practice can potentially reduce caregiver distress and assist in promptly recognizing and treating delirium in the PICU.

## Supplementary material

10.2196/53120 Multimedia Appendix 1 Focus group guide for health care professionals and parents.

## Abbreviations

BCCH: BC Children’s Hospital
COREQ: Consolidated Criteria for Reporting Qualitative Research
PICU: pediatric intensive care unit
REDCap: Research Electronic Data Capture
