# Supplementary material for: Pediatric Delirium Educational Tool Development With Intensive Care Unit Clinicians and Caregivers in Canada: Focus Group Study
Source: JMIR Pediatr Parent. 2023 Dec 11;6:e53120. doi: 10.2196/53120 (PMC10734902; doi:10.2196/53120)
Supplement: Multimedia Appendix 1 [file pediatrics-v6-e53120-s001.docx]

# Supplemental Material 1. Focus group guide for caregivers and healthcare professionals.

**Facilitator Script for Each Session**:

“Good morning/afternoon/evening, thank you for taking the time to join us today. My name is __________ and I am a <role & institution> and I welcoming you to this session today because you have lived experience as caregiver of a child who recently received intensive care, or are a healthcare professional with expertise in pediatric delirium, both of which will provide invaluable insight for developing a pediatric delirium educational tool. To make sure we are all on the same page, I will start with a short overview of what I mean by delirium. Delirium is a form of brain dysfunction that is characterized by a sudden onset, inattention, and/or confusion, which results in the patient behaving in an atypical manner, which is a difficult experience for the patient and their family. Educational strategies, such as brochures, may help family members recognize delirium symptoms and deviations from their child’s typical behaviors and assist doctors in quick administration of treatment. Our overall goal is to investigate how we can develop effective education tools for caregivers whose child is experiencing delirium in the pediatric intensive care unit at BC Children’s Hospital (BCCH).

To achieve this goal, we first need to learn more about the potential information that should be included in our tool and obtain potential design strategies that can ensure effective use by parents. We will use this information to design a delirium education tool for parents whose child is experiencing delirium.

Your input is essential to our study, and we genuinely appreciate your participation today. As outlined in the consent form, we are audio recording these sessions. However, if anyone is uncomfortable with participating or being recorded, you may withdraw from the study at any time by exiting the session – use the red “Leave” button on the bottom right of the Zoom window.”

## Participant introduction and community agreements for each session:

- Ensure each research team member present briefly introduces themselves and their role on the project.
- Ask each participant to briefly introduce themselves.
- “Please remember we’re here to exchange opinions and ideas, so please respectfully state what you think. We are not assessing your performance or your knowledge on the topics discussed.”
- “To reduce unnecessary background noise, please turn off or silence cell phones, as well as mute your microphone when not speaking. We ask that only one person speaks at a time. When you wish to speak, wait for the current speaker to finish their point, and then unmute your microphone to comment. Alternatively, please use the raise hand feature in Zoom to indicate you would like to speak. I might also call on you directly for your opinions and thoughts.”
- “If you need to, feel free to take a break or sign off the session, if necessary.”
- “Please remember that this is a closed space with confidentiality in mind.”
- “Our session ends at [insert time] so let’s begin.”

Enable Zoom UBC Cloud recording.

**Introduce the specific purpose of the current workshop (1 or 2):**

1. Participants will be asked to speak to how to develop an education tool (e.g., sections that should be included, definitions, etc.). Then they will be asked to provide their opinions and ideas on the design requirements needed to develop delirium educational materials.
2. Participants will provide feedback on mock-ups of the educational materials that will help guide the next design iteration and provide suggestions for refinement.

**Tasks for Session 1:**

- Prompt discussion on how participants are typically told about delirium PICU associated delirium while at BCCH.
- Query participant opinions on how best to present delirium educational information clearly and effectively.
- Discuss design strategies that could ensure effective utilization of the tool by parents.
- Look at examples of delirium educational tools for adult populations to elicit additional strategies to ensure effective delirium education.

**Questions for Session 1:**

1. Can you please tell us about how you were provided with (or provide) delirium education in the pediatric intensive care unit (PICU)?
   1. Probe. If delirium was not discussed, what would you want your doctor and medical team to tell you about it?
   2. Probe. If delirium was discussed, was the format of the education useful?
2. What type of educational tools/instruments have you used previously?
   1. Probe. Can you tell me about your current experience with these tools/instruments?
   2. Probe. If you do not have any experience, can provide suggestions for developing such a tool (broadly speaking)?
3. To be provided with delirium education clearly and effectively, what type of information would you want to have available?
   1. Probe: Would there be certain sections/headers that we should include this information under?
4. Screen share examples of delirium education tools over Zoom. Discuss what sections and formats were useful in terms of providing delirium education.
   1. Probe: Are there sections that could be improved (or added)?
   2. Probe: Are there gaps in education that are not being provided?
5. Were there any final thoughts on anything that we discussed here today?

**End Session 1 with the following reminders:**

- Summarize main points discussed throughout the meeting.
- There will be one additional focus group; our team will be sending you an e-mail shortly to set-up our next meeting. If you return, you will be asked to give your feedback on our developed education tool, which will guide our final design. Given that the final session builds on the first, we would greatly appreciate your continued participation. However, you do not have to participate in the final session.
- Thank you so much for attending and sharing your thoughts and opinions with us today.

**Tasks for Session 2:**

1. Brief review of previous session’s discussion and conclusions.
2. Outline the purpose of the session: obtain additional requirements and feedback to further improve our current design.
3. Screen share the education tool prototype over Zoom.
4. Describe each section to participants to acquire detailed feedback and suggestions for potential improvements.
5. Ask participants to additional design ideas.

**Questions for Session 2:**

1. Can you please share what sections, if any, of the educational tool were especially clear and concise?
   1. Probe: Were any sections of the tool unclear? If so, how could they be improved?
2. Now that we have reviewed the document, are there additional strategies that might help to ensure that this information is presented clearly and effectively?
3. Were there any final thoughts on anything that we discussed here today?

## End Session 2 with the following reminders:

## Summarize main findings.

- As stated in the consent form, the main study findings will be published in academic journal articles but these data will be de-identified to protect your privacy and security. As such, we’ll share the final project report and findings (via email) about 6 months after we finish the project.

## If there are no further questions and/or comments, that concludes our focus group meeting. Thank you so much for attending and sharing your thoughts and opinions with us today.
